# Solution-Processed One-Dimensional ZnO@CdS Heterojunction toward Efficient Cu_2_ZnSnS_4_ Solar Cell with Inverted Structure

**DOI:** 10.1038/srep35300

**Published:** 2016-10-13

**Authors:** Rongrong Chen, Jiandong Fan, Chong Liu, Xing Zhang, Yanjiao Shen, Yaohua Mai

**Affiliations:** 1 Institute of Photovoltaics, College of Physics Science and Technology, Hebei University, Baoding, 071002, China; 2 Institute of New Energy Technology, College of Information and Technology, Jinan University, Guangzhou, 510632, China

## Abstract

Kesterite Cu_2_ZnSnS_4_ (CZTS) semiconductor has been demonstrated to be a promising alternative absorber in thin film solar cell in virtue of its earth-abundant, non-toxic element, suitable optical and electrical properties. Herein, a low-cost and non-toxic method that based on the thermal decomposition and reaction of metal-thiourea-oxygen sol-gel complexes to synthesize CZTS thin film was developed. The low-dimensional ZnO@CdS heterojunction nano-arrays coupling with the as-prepared CZTS thin film were employed to fabricate a novel solar cell with inverted structure. The vertically aligned nanowires (NWs) allow facilitating the charge carrier collection/separation/transfer with large interface areas. By optimizing the parameters including the annealing temperature of CZTS absorber, the thickness of CdS buffer layer and the morphology of ZnO NWs, an open-circuit voltage (V_OC_) as high as 589 mV was obtained by such solar cell with inverted structure. The all-solution-processed technic allows the realization of CZTS solar cell with extremely low cost.

Global energy consumption has increased dramatically with population growth and industrialization development, which gradually swallowed up the non-renewable resources^1^. In this regard, solar cell is considered as one of the likely key solutions to satisfy the growing global energy demand with respect to its harmlessness, environmental protection and sustainable utilization of energy. At present, 89% of the photovoltaic module market is captured by the crystalline silicon solar cells^2^. However, the expensive and cumbersome solar cells technology limits the cost reduction of the crystalline silicon solar cells. Recently, thin film solar cells including amorphous silicon, Cu(In,Ga)Se_2-x_S_x_ (CIGS) and CdTe, began to set foot in the market due to its low price^3^,^4^,^5^. However, the preparation of these solar cells have suffered from either severe preparation condition with high vacuum and high temperature, and/or the limited reserve of inorganic materials on the earth. Aside from this, its toxicity also limits their large production^6^,^7^.

As an excellent representative of the third generation solar cells, copper zinc tin sulfur Cu_2_ZnSnS_4_ (CZTS) received extensive attention, which gives the credit to its environmentally friendly features, rich content in the earth’s crust and decent photoelectric properties^8^,^9^,^10^. It is well known that the bandgap width of CZTS is ~1.5 eV, which matches well with the other semiconductor materials in solar cell^11^,^12^,^13^,^14^. Apart from these, its great light absorption coefficient (10^4^ cm^−1^ in visible light region) is favorable to the light absorbance in UV-vis range. Consequently, CZTS solar cell photoelectric conversion efficiency (PCE) increased from 0.66% in 1996 to 6.7% in 2008, then to 12.6% in 2012 by Solar Frontier company^15^,^16^,^17^,^18^,^19^. Unquestionably, CZTS films will become another promising material after CIGS series materials in the near future with continual improvement in preparation technology.

At present, CZTS thin films can be obtained by various approaches including vacuum thermal evaporation^20^, electron beam evaporation^21^, sputtering^22^, spray pyrolysis^23^, and pulsed laser deposition^24^. However, these approaches are neither versatile nor particularly low-cost toward the realization of large area devices due to the low yields and growth rate they provide and the controlled atmospheres and relatively high energies they require. In this scenario, the solution-processed CZTS film exhibits relatively outstanding advantage owing to its simplicity and potential for large scale and low cost production^25^,^26^. Likewise, a low dimensional heterojunction arrays allow the concurrence of high efficiencies of charge carrier transport with large interface areas for charge separation and/or transfer with the surrounding species^27^,^28^. Recently, a solar cell with inverted structure combining with the heterojunction nano arrays and solution-processed CZTS or CIGS thin film was demonstrated to exhibit an excellent photovoltaic performance in contrast to a planar film device owing to its enhanced light transmittance toward high charge collection efficiency^29^,^30^,^31^,^32^. However, the detailed deposition technology and the interfacial engineering still need further optimization toward highly effective solar cell with inverted structure.

Herein, a low dimensional ZnO@CdS nano heterojunction arrays were synthetized by aqueous solution method as the window layer and buffer layer in the solar cell, respectively. A low-cost and non-toxic method that based on the thermal decomposition and reaction of metal-thiourea-oxygen sol-gel complexes to synthesize CZTS thin film was developed. The as-obtained solar cell with inverted structure of FTO/ZnO@CdS NWs/CZTS/Ag exhibited outstanding photovoltaic performance with relatively high *V*_*OC*_ of 589 mV.

## Results

### Preparations and characterizations of ZnO@CdS NWs thin films

**Figure 1:**
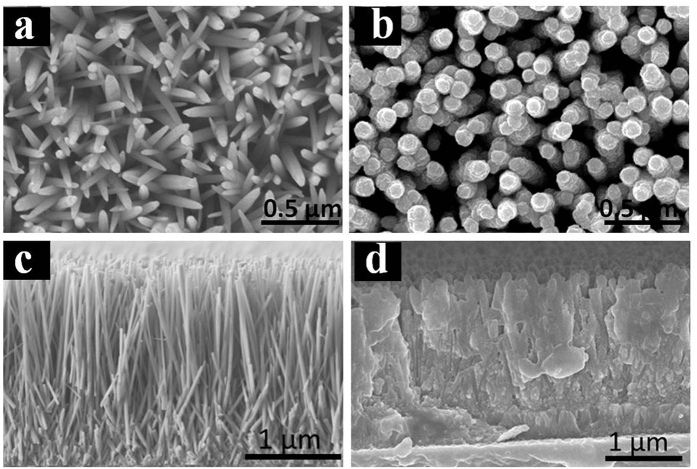
Top-down SEM images of (a) bare ZnO NWs and (b) ZnO@CdS NWs, (c,d) are the corresponding cross-sectional SEM images of (a,b), respectively.

Here, the ZnO NWs were prepared via a hydrothermal method at 90 °C keeping a short reaction time. It is well known that the controllable morphology of ZnO NWs can be obtained by tuning the deposition time^33^. Accordingly, an array of hexagonal ZnO NWs with 2 μm in length and 50–60 nm in diameter was produced as shown in Fig. 1a,c. It is worthy of noting that the morphology of ZnO NWs including the length and diameter was proven to be a critical factor to dominate the photovoltaic performance of the NWs-based solar cell^33^. The detailed influence of various lengths of ZnO NWs on the photovoltaic property will be elucidated afterward. Here, we employed the ZnO NWs with the same morphology as shown in Fig. 1a,c to fabricate the solar cell. Likewise, it is well known that CdS as a buffer layer in the solar cell plays a crucial role in determining the photovoltaic performance^34^. Herein, the CdS shell was coated on ZnO NWs by nanocrystal layer deposition (NCLD) technique^35^ at room temperature. As shown in Fig. 1b,d, a uniform CdS shell layer has formed on the ZnO NWs as a buffer layer. The effect of CdS buffer layer on the photovoltaic property of solar cell will be further discussed later.

### Preparations and characterizations of CZTS thin films

**Figure 2:**
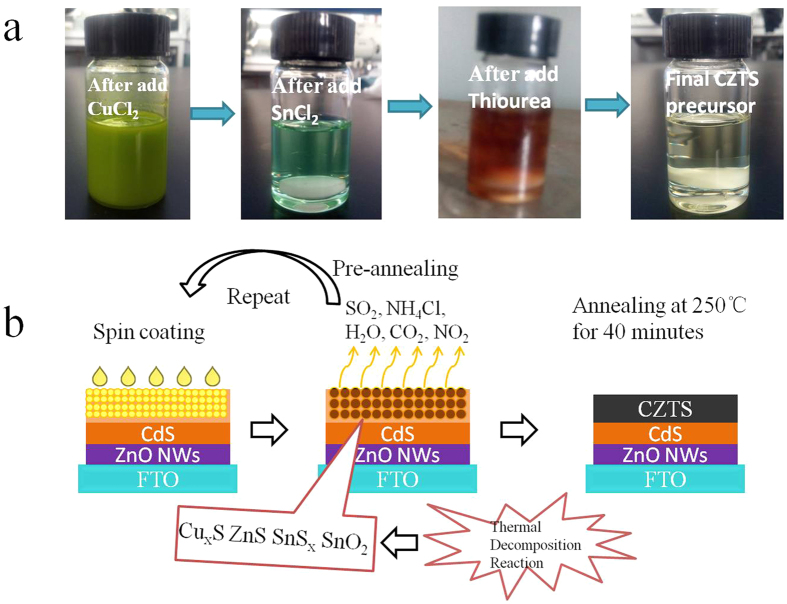
(**a**) Photographs of the precursor solutions for the CZTS preparation in each step. Note that the different colors in solution. (**b**) Illustration of the formation of the CZTS thin films via thermal decomposition and reaction by the sol–gel route.

To finalize the NWs-based solar cell, a CZTS precursor solution was spin-coated on the as-prepared ZnO@CdS NWs. Prior to the spin coating process, the CZTS precursor solution was prepared by adding different precursors into the solvent step-by-step as shown in Fig. 2a. The change from deep-blue suspension to a very light green-yellow clear solution after the first two-steps indicates the reduction of Cu^2+^ to Cu^+^ (Equation 1), accompanied by solvation/stabilization of metal ions by DMSO and/or chloride anions. Note that the final light-yellow solution suggests that little Cu^2+^ remains after adding thiourea, which can be explained by the formation of CuCl_4_^−^ (a yellow species) while the unreacted Cu^2+^ in high Cl^−^ containing solution^36^. Precursor solution was then used to spin on the ZnO NWs, it was found that some gases were generated, i.e., NH_4_Cl, CO_2_, SO_2_ and H_2_O while pre-annealing the as-prepared film at 200 °C (Equations 2 and 3).


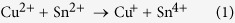



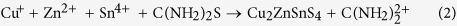



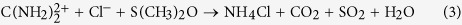


The as-obtained CZTS thin film was afterward annealed with an aim of further improving its crystallinity (Fig. 2b). As shown in Fig. S3, it turns out that the crystallinity of CZTS thin film is inferior without annealing process. The crystal quality has been improved with increased annealing temperature. However, severe cracks were found while increasing the annealing temperature up to 400 °C, which might be associated with the solvent evaporation and/or the crystal shrink during the annealing process.

**Figure 3:**
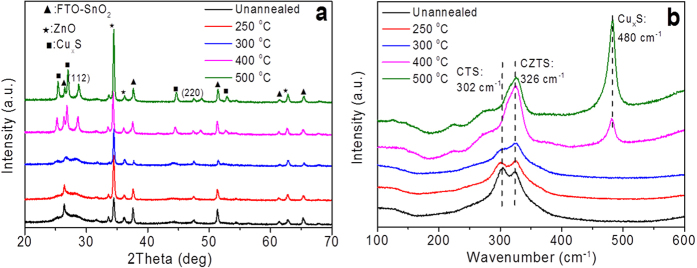
(**a**) XRD patterns of CZTS films prepared by coating the precursor solution on ZnO@CdS films at various annealing temperatures; (**b**) The corresponding Raman spectra of CZTS films.

To further study the structure evolution of CZTS thin film after post-thermal treatment. We carried out the XRD and Raman characterizations for the film annealed at different temperature (Fig. 3). As shown in Fig. 3a, it is clearly shown that the peaks ascribed to hexagonal ZnO and FTO substrate were found in all cases. The peaks assigned to (112) and (220) tent to appear when the annealing temperature increased up to 250 °C, which suggests the as-prepared CZTS thin film remain amorphous below 250 °C. The crystallinity of CZTS thin film has been significantly enhanced when the annealing temperature was above 400 °C. However, the peaks associated with binary phase of Cu_x_S appeared while improving the crystallinity of CZTS thin film, which usually gave rise to the inferior photovoltaic performance of CZTS solar cell^37^. As shown in Fig. 3b, the Raman mode at around 326 cm^−1^ is associated with the as-prepared kesterite CZTS thin film, which was in consistent with the previous reports^38^,^39^. Meanwhile, the mode intensity increased with the increased annealing temperature. Again, the Raman mode ascribed to the binary phase of Cu_2_S was detected in the case of annealing temperature up to 400 °C. The results further certified the presence of binary phase of Cu_x_S. Unexpectedly, we just detected quite small amount of binary phase of Cu_2_S while spinning coating the CZTS precursors on planar FTO substrate as shown in XRD patterns and Raman spectra (Fig. S1). We assumed that the presence of CdS buffer layer may lead to the formation of Cu_2_S phase. Particularly, the excess of S^2−^ in the interface may react with the Cu^2+^ species on the surface of CZTS thin film, which gives rise to the formation of Cu_2_S phase in the interfacial domain while annealing the ZnO@CdS NWs/CZTS thin film.

**Figure 4:**
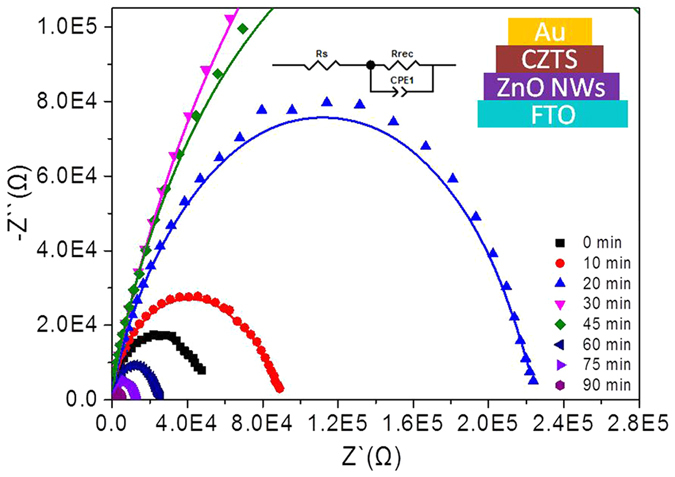
Nyquist plots of the impedance data of the ZnO NWs/CZTS heterojunction film in the dark. The solid lines are the fitting results based on the equivalent circuit model shown in the inset.

Electrochemical impedance spectroscopy (EIS) was used to evaluate the resistance for charge carrier transfer between ZnO NWs and CZTS film. Figure 4 displays the Nyquist plots of the heterojunction films by ZnO NWs with different length grown for different time in the range of 0–90 min. The results can be accordingly fitted with the equivalent circuit model shown in the inset, which is consists of a resistor R_s_, a constant phase element (CPE) and a charge transfer resistance R_rec_. Here, the R_rec_ is usually dependent on the charge transport process at the interface. The CPE can be defined as CPE-T and CPE-P, which are associated with the interfacial capacitor and an idea capacitor, respectively. The corresponding parameters by fitting from Nyquist plots are shown in Table 1. It clearly demonstrated that the R_s_ tent to decrease and then increase with the enhanced length of ZnO NWs, whereas the R_rec_ displayed an inverse tendency that firstly increased and then decreased. The interface based on ZnO NWs grown for 30 min was demonstrated to have the lowest R_s_ and highest R_rec_. This suggests that the presence of ZnO NWs with suitable length (2 μm) was able to improve the interfacial contact between the ZnO NWs and CZTS film, and thus tune the charge transfer resistance R_rec_.

### Photovoltaic performances of CZTS solar cells

**Figure 5:**
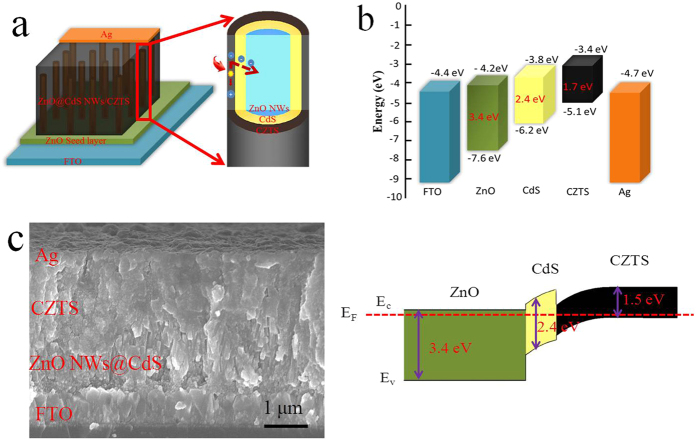
Schematic diagram of (a) CZTS solar cell based on ZnO@CdS NWs with inverted structure (FTO/ZnO@CdS NWs/CZTS/Ag); (b) The bandgap alignment of each layer in CZTS solar cell; (c) Cross-sectional SEM image of the as-cleaved device (FTO/ZnO@CdS NWs/CZTS/Ag).

Figure 5a,b show a typical band diagram for the CZTS cell with inverted structure^40^,^41^. The wider band gaps of the ZnO and CdS allow the majority of photons to be absorbed in the narrow-gap p-type absorber (CZTS). Electron-hole pairs generated by the photons in the CZTS absorber are separated by the built-in electric field in the p-n junction and contribute to the cell’s photocurrent. The V_OC_ should be determined by the built-in potential. In this scenario, higher absorber band gaps should yield higher V_OC_ and lower J_SC_. Electron-affinity difference at the buffer/absorber hetero-interface would result in a conduction band discontinuity.

**Figure 6:**
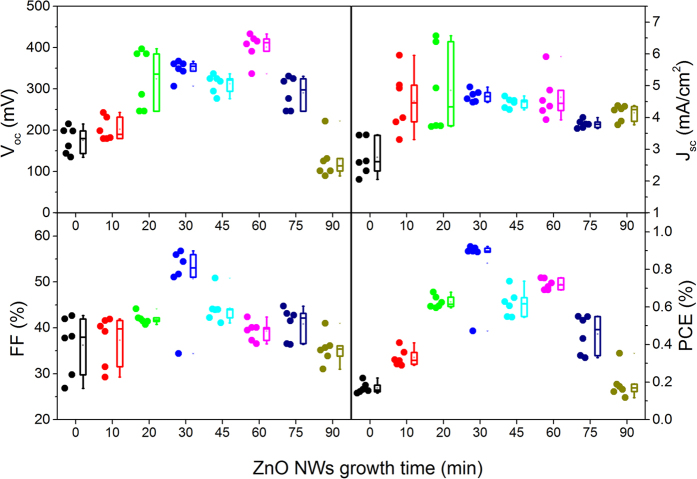
Photovoltaic performance of the as-prepared solar cells as a function of the length of ZnO NWs.

Here, we fabricated the ZnO NWs-based CZTS solar cells as shown in Fig. 5c. As mentioned previously, the morphology of ZnO NWs has a significant influence on the photovoltaic performance of NWs-based solar cell. We have explored the effect of the length of ZnO NWs that synthesized hydrothermally by different time (0–90 min) on the photovoltaic properties of ZnO NWs-based CZTS solar cell. As shown in Fig. 6, it turns out that the ZnO NWs grown for 30 min (the length is around 2 μm) have the best photovoltaic performance. It should be noted that the PCE of the planar ZnO-based cell was only around 0.2%, which was inferior to the ones with ZnO NWs. Clearly, the lower J_SC_ resulted in the decreased PCE. We inferred to the presence of ZnO NWs was favorable to load more CZTS nanoparticles and thereby enhance the photo absorbance. Likewise, the one dimensional ZnO arrays allow the concurrence of high efficiencies of charge carrier transport with large interface areas for charge separation and/or transfer with the surrounding species. Unlike the ZnO NWs based dye-sensitized solar cell, CZTS has great light absorption coefficient in comparison to the widely-used dye. The obtained results concluded that the ZnO NWs with 2 μm in length was more than enough to load substantial CZTS absorber for light soaking. As evidence, the longer NWs over 2 μm were demonstrated to quench the photovoltaic property that mainly caused by the recombination of electro-hole pairs, which was unlike the case of dye-sensitized solar cells^33^.

**Figure 7:**
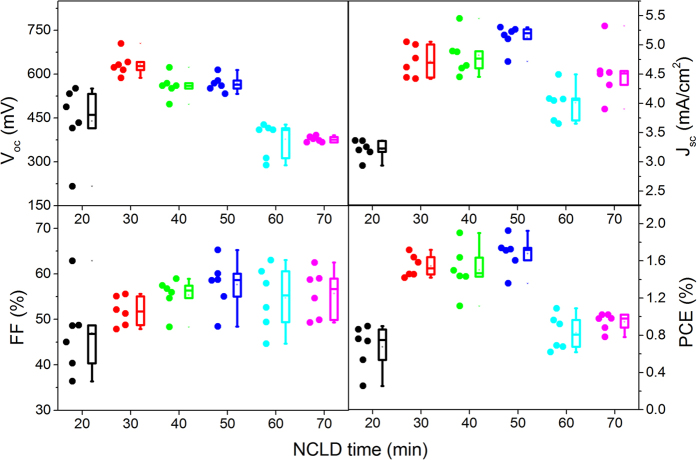
Effect of the thickness of CdS buffer layer on the photovoltaic performance of CZTS solar cells.

In the beginning, we fabricated the solar cell as follows: FTO/ZnO NWs/CZTS/Ag. The as-prepared device displayed poor photovoltaic performance due to the non-existence of the buffer layer in above solar cell (Fig. S3b). It is well known that the introduction of a buffer layer into a CZTS solar cell was favorable to enhance the device performances by avoiding undesirable shunt path and widening the depletion width at the p−n interface that extend electric field in absorber layer and minimize the collection loss by tunneling and recombination^35^. Here, we prepared the CdS layer by solution-processed NCLD technique. It is reasonable that the XRD peaks of CdS were not detected after the CdS deposition as shown in Fig. S3a, which can be attributed to the thinner thickness of CdS that was estimated to be in the scale of nanometers. Nevertheless, the absorption edge had an obvious red-shift from 380 nm to 510 nm after coating CdS layer on ZnO NWs (Fig. S4). The absorbance edge at 510 nm corresponding to the band gap energy of bulk CdS (2.4 eV) suggested that the formation of CdS layer. Expectedly, the photovoltaic performances of the device including open voltage (V_OC_) and short-circuit (J_SC_) has been substantially enhanced after coating the CdS buffer layer between ZnO NWs and CZTS thin film (Fig. S3b), although the fill factor (FF) has a slightly decreased, which give rise to the improved power conversion efficiency (PCE) by a factor of 2.5. With respect to the effect of CdS thickness on the photovoltaic performance of device, we obtained different thickness of CdS by optimizing the deposition time. As shown in Fig. 7, it was found that the PCE was firstly increased and then decreased progressively as the function of CdS thickness. The CdS layer deposited for 50 min was demonstrated to be the best thickness toward highest PCE.

As mentioned previously, the post-annealing process dominated the morphology and crystallization of CZTS thin film. The influence of the CZTS annealing temperature on the photovoltaic performances was investigated by means of measuring the current density−voltage (J−V) curves for each set of experimental conditions under AM 1.5 (100 mV/cm^2^). Table 2 compares the detailed parameters obtained from the J−V measurements. It should be mentioned that the ZnO NWs with 2 μm in length and a CdS buffer layer deposited by NCLD for 50 min were employed to fabricate the solar cell. The best performances of solar cell were observed from CZTS thin film annealed at 250 °C. Moreover, we further optimized the ratio effect of Zn/Sn in the precursor solution on the photovoltaic performances. The optimized results demonstrated that the best performances can be obtained while the ratio of Zn/Sn was around 1.1. The corresponding cell yielded a J_SC_ of 7.07 mA/cm^2^, a V_OC_ of 589 mV, and a FF of 0.54, resulting in a PCE of 2.27%. The obtained PCE is comparable to the previous report that has the highest PCE in this type solar cell with inverted structure^42^.

Theoretically, a reduced minority carrier flux across the junction boundary in solar cell will produce a smaller V_OC_ in a planar heterojunction cell^34^. The vertically aligned NWs give a special geometric that causes the different junction area from planar thin film. The correctional V_OC_ of a NWs-based solar cell can be written as follows (Equation 4) refer to its geometric factors:


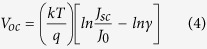


where *k* is Boltzmann constant, *T* is the absolute temperature, *q* is the charge of electron, *J*_*0*_ is the reverse saturation current density, and *γ* is the ratio between the junction area of a NW arrays and that of a planar thin film, which can be written as follows: *γ = S*_*NWs*_*/S*_*Planar*_* = 2πrhρ*_*NWs,*_ Herein, r = 80∼100 nm, h ≈ 2000 nm, *ρ*_*NWs*_ ≈ 10^11^cm^−2^. These parameters will yield a V_OC_ loss of 90∼100 mV. The obtained V_OC_ loss can elucidate the increased V_OC_ value in the NWs-based solar cell in comparison to the planar one that usually has a V_OC_ of around 500 mV^43^,^44^,^45^.

## Discussion

In comparison to the unannealed sample, the improved crystallinity of CZTS after annealing process can account for the enhanced photovoltaic properties. Nevertheless, severe cracks were found when the annealing temperature rose up to 300 °C as discussed above, which might result in the deteriorative PCE parameters. It is should be mentioned that, although the CZTS thin film annealed at 500 °C has been thoroughly crystalized, we have to employed the film annealed at 250 °C, which is associated with the sever cracks and the used CdS buffer layer that could not endure the post-annealing process at high temperature. The alternative buffer layer would further improve the PCE of the CZTS solar cell with such inverted structure.

In summary, we report a low-cost and non-toxic method that based on the thermal decomposition and reaction of metal-thiourea-oxygen sol-gel complexes to synthesize CZTS thin film. The low dimensional NWs enable the realization of efficient charge carrier collection/separation/transfer with large interface areas. Moreover, the presence of CdS buffer layer with controllable thickness allows substantially improving the photovoltaic performance. By optimizing the technical parameters, an effective CZTS solar cell based on ZnO@CdS heterojunction NWs can be obtained with a PCE of 2.27% and a high *V*_*OC*_ of 589 mV. Such all-solution-processed CZTS solar cell with inverted structure is demonstrated to be capable of extremely reducing the cost. Further studies toward higher efficiency, e.g., enhancing the crystallization of CZTS films via suitable S/Se treatments, swapping the CdS buffer layer by alternative one that can endure higher annealing temperature, are underway.

## Methods

### Materials

All reagent were of analytical grade and used without any further purification. Zinc acetate dihydrate (Zn(OAc)_2_·2H_2_O, 99%), diethanolamine (C_4_H_11_NO_3_, 99%), dimethylcarbinol (C_3_H_8_O, 99.7%), zinc nitrate hexahydrate (Zn(NO_3_)_2_·6H_2_O, 99%), hexamethylenetetramine (C_6_H_12_N_4_, 99%), polyethyleneimine ((CH_2_CH_2_NH)n,), ammonium hydroxide (28 wt% NH_3_ in water, 99.99%), cadmium sulfate (CdSO_4_, 99%), thioacetamide (C_2_H_5_NS, 99%), cupric chloride dihydrate (CuCl_2_·2H_2_O, 99%), tin dichloride dihydrate (SnCl_2_·2H_2_O, 98%), zinc chloride (ZnCl_2_, 98%), thiourea (CH_4_N_2_S, 99%), dimethylsulfoxide (DMSO, anhydrous) and sodium chloride (NaCl, 99.5%) were used. Prior to the material deposition, F-doped tin oxide (FTO) coated soda lime glass (SLG) substrates were ultrasonically cleaned for 10 minutes with deionized water, acetone, and ethanol, respectively.

### Growth of ZnO NWs

ZnO NWs were obtained by a low-cost, high-yield, and large-area hydrothermal process. First, the ZnO seed precursor solution was prepared by adding 0.5 M Zn(OAc)_2_ and 0.5 M diethanolamine into dimethylcarbinol with stirring for an hour at 60 °C. Then as-prepared ZnO seed precursor solution was spin coated on the conductive side of the FTO substrate, and finally annealed in air at 400 °C for 60 minutes. And then the seeded substrate was placed inside a 500 ml beaker containing an aqueous solution with 20 mM Zn(NO_3_)_2_, 15 mM hexamethylenetetramine (HMTA), 4 mM polyethyleneimine (PEI), and 24 mM ammonium hydroxide. The FTO substrate covered ZnO seed was kept for 30–90 minutes inside a water bath at 90 °C^33^. Consequently, vertically aligned ZnO NWs with different length were obtained. The as-prepared ZnO NW arrays were afterward rinsed with deionized water and ethanol, finally annealed in air at 400 °C for 60 minutes.

### Deposition of the CdS Buffer Layer

The ZnO NWs were modified by CdS deposition using nanocrystal layer deposition (NCLD) techniques^35^. Briefly, the CdS was deposited with a mixed solution of 20 mM CdSO_4_ solution and 20 mM thioacetamide (TAA) aqueous solution for 20–70 minutes at room temperature.

### Fabrication of the ZnO@CdS/CZTS photovoltaic device

The CZTS precursor solution was prepared by adding the precursors into the solvent step-by-step as shown in Fig. 1. In particular, first, 10 mL dimethylsulfoxide (DMSO) was added to a vial containing 0.5 M CuCl_2_·2H_2_O, resulting in a deep blue suspension due to poor solubility of CuCl_2_·2H_2_O in DMSO. Second, the precursor of SnCl_2_·2H_2_O (0.25 M) was added into the vial, and the deep blue suspension gradually changed to clear light green solution after overnight stirring. Afterward, adding the third precursor of ZnCl_2_ (0.25 M) into the solution, which resulted in the color changed to lighter green, with a yellow tint. Then, 1.5 M thiourea was added, and a transparent and colorless solution was obtained after thiourea completely dissolved. Finally, 0.1 M NaCl was added into the solution. The transparent and colorless precursor solution was spin-coated onto the as-prepared ZnO@CdS NW samples at 3000 rpm for 30 seconds. The obtained samples were then placed on a 200 °C preheated hot plate for 2 minutes. This coating–drying cycle was repeated six times with an aim of obtaining controllable thickness of CZTS thin film. After the cycles, the as-prepared samples were immediately transferred onto a 250 °C preheated hot plate and maintained at this temperature for 40 minutes. After heat treatment, the devices were allowed to cool to room temperature in an air environment.

### Characterization

The crystal structure was characterized by Bruker D8 Advance X-ray diffractometer (XRD) with Cu Kα radiation at 40 kV and 40 mA. Field-emission scanning electron microscopy (SEM) was used to characterize the morphology of the obtained thin film. Both top-down and cross-sectional views were obtained using a JEOL JSM-7500F. A double beam spectrophotometer (U-4100, Hitachi) equipped with an integrated sphere was used for the UV-vis transmission measurements in the range from 340 to 1100 nm. Impedance spectroscopy (IS) measurements were carried out with use of an impedance analyzer (Zahner, Zennium) under dark conditions at 0 V, applying a 10 mV ac sinusoidal signal over the constant applied bias with the frequency ranging from 1 MHz to 0.1 Hz. Current-voltage (J-V) characteristics of CZTS solar cells were measured using a semiconductor device analyzer (Keithley 2601B) and a SAN-EI solar simulator (XES-100S1) with an AM 1.5 G spectrum. The illumination power on the sample was adjusted to 1000 W m^−2^ using a certified reference solar cell (RS-ID-4). The scan rate was fixed to 0.15 V/s. A black mask with an aperture (9 mm^2^) was placed on the top of the device to control the effective electrode area. Raman scattering spectroscopy was performed using a LabRAM HR evolution of Horiba Raman scattering system with a 100X magnification lens and in the backscattering configuration. All the Raman scattering measurements were performed using an excitation wavelength of 532 nm.

## Additional Information

**How to cite this article**: Chen, R. *et al*. Solution-Processed One-Dimensional ZnO@CdS Heterojunction toward Efficient Cu_2_ZnSnS_4_ Solar Cell with Inverted Structure. *Sci. Rep.* **6**, 35300; doi: 10.1038/srep35300 (2016).

## Supplementary Material

Supplementary Information

## Figures and Tables

**Table 1:** Parameters determined by EIS measurements for the ZnO NWs/CZTS heterojunction film.

ZnO NWs growth time (min)	Rs (Ω·cm^2^)	Rrec (Ω·cm^2^)	CPE-T (F/cm^2^)	CPE-P (F/cm^2^)
0 min	39.97	44754	4.35E-08	0.902
10 min	39.28	78444	3.41E-08	0.872
20 min	27.21	210410	2.86E-08	0.853
30 min	19.72	565250	2.38E-08	0.848
45 min	35.69	343220	4.38E-08	0.893
60 min	37.98	23965	6.39E-08	0.886
75 min	39.81	12006	1.20E-07	0.868
90 min	42.15	5160	1.95E-07	0.852

**Table 2:** Photovoltaic properties of the fabricated solar cells, Namely, short-circuit current (*J*_*SC*_), open-circuit voltage (*V*_*OC*_), fill factor (*FF*), and power conversion efficiency, as functions of the CZTS crystallization temperature.

Temperature (°C)	J_sc_ (mA/cm^2^)	V_oc_ (mV)	FF (%)	PCE (%)
Unannealed	4.78	319	47	0.73
250	7.07	589	54	2.27
300	4.42	397	57	1.00
400	3.40	67	27	0.06
500	0	0	0	0
