# Supplementary material for: Solution-Processed One-Dimensional ZnO@CdS Heterojunction toward Efficient Cu2ZnSnS4 Solar Cell with Inverted Structure
Source: Sci Rep. 2016 Oct 13;6:35300. doi: 10.1038/srep35300 (PMC5062431; doi:10.1038/srep35300)
Supplement: Supplementary Information [file srep35300-s1.doc]

Supporting Information

**Solution-Processed One-Dimensional ZnO @ CdS Heterojunction toward Efficient Cu_2_ZnSnS_4_ Solar Cell with Inverted Structure**

*Rongrong Chen^1^, Jiandong Fan^1,2^*,Chong Liu^1^, Xing Zhang^1^, Yanjiao Shen^1^, Yaohua Mai^1,2^*
^1^Institute of Photovoltaics,College of Physics Science and Technology, Hebei University, Baoding, 071002, China*

*^2^Institute of New Energy Technology, College of Information and Technology, Jinan University, Guangzhou, 510632, China*

Corresponding Authors

Phone: +86 312 5077382

Fax: +86 312 5077382

*E-mail: (J. F.) [jdfan@jnu.edu.cn](mailto:jdfan@jnu.edu.cn);

*E-mail: (Y. M.) [yaohuamai@jnu.edu.cn](mailto:yaohuamai@jnu.edu.cn).

Figure S1 (a) XRD patterns of CZTS films prepared by coating a precursor solution on FTO substrate with various anneal temperatures; (b) The corresponding Raman spectra of CZTS films.

Fig.S1 displays the evolution of XRD and Raman spectra of CZTS films prepared directly on FTO substrate with sol-gel method at different annealing temperature. It is found that an increase of diffraction peak intensity with rising the annealing temperature due to the improved crystallinity of CZTS thin film. As shown in Fig. S1b, the Raman spectra of CZTS films exhibits two peaks near 285 cm^−1^ and 335 cm^−1^, both of which are assigned to be kesterite CZTS. And Raman modes of the other secondary phases were not been detected.

Figure S2 Top-down SEM images of CZTS films prepared by coating a precursor solution on FTO substrate with various anneal temperatures: (a).Unannealed, (b).250 ^o^C, (c).300 ^o^C, (d). 400 ^o^C, (e). 500 ^o^C .

As shown in Fig. S2, the crystallinity of CZTS thin film was improved with rising the annealing temperature. We did not found any cracks except for some pinholes, whereas severe cracks were found on the structure of FTO/ZnO@CdS NWs/CZTS.

Figure S3 Top-down SEM images of CZTS films prepared by coating a precursor solution on ZnO NWs with various annealing temperatures: (a).Unannealed, (b).250 ºC, ( c).300 ºC , (d). 400 ºC, ( e). 500 ºC.

Figure S4. (a) XRD of bare ZnO NWs and ZnO@CdS NWs; (b) *I-V* curves of the solar cell (FTO/ZnO@CdS NWs/CZTS/Ag) and the device without CdS buffer layer.

The phase and structure of thin film were confirmed by XRD. As shown in Fig.S3a, bare ZnO NWs exhibits a sharp peak at 34.4°, which corresponds to the (002) planes of hexagonal crystal structure (PDF#36-1451). The vertically aligned ZnO NWs grown along <001> direction were certified by the obtained XRD patterns. After NCLD deposition, CdS peaks were not observed from XRD characteristic, which is results from the CdS buffer layer synthesized by NCLD is extraordinarily thin (～20 nm). Fig. S3b displays how the presence of CdS buffer layer affects the photovoltaic performance of CZTS solar cell.

Figure S5 Absorption spectra of the ZnO NWs, ZnO@CdS NWs, ZnO@CdS/ CZTS NWs.

Fig. S5 displays the absorption evolution of the bare ZnO NW arrays and after the deposition of CdS and CZTS layers via sol-gel methods in the range of 350–1100 nm, respectively. The spectrum of bare ZnO NWs shows a clear band-edge absorption at 380 nm, corresponding to the band gap energy of ZnO (3.3 eV). The absorption edge was observed at 510 nm in the case of CdS coating on ZnO NWs, corresponding to the band gap energy of bulk CdS (2.4 eV). After the deposition of CZTS thin film, the absorption edge has a red shift to 850 nm, which is associated with the band gap energy of bulk CZTS(1.4–1.5 eV).

Figure S6 Effect of the ratio of Zn/Sn on the photovoltaic performance of CZTS solar cells.
